# Performance of the Dutch SF-36 version 2 as a measure of health-related quality of life in patients with rheumatoid arthritis

**DOI:** 10.1186/1477-7525-11-77

**Published:** 2013-05-08

**Authors:** Peter M ten Klooster, Harald E Vonkeman, Erik Taal, Liseth Siemons, Lidy Hendriks, Alphons J L de Jong, Ellen A J Dutmer, Piet L C M van Riel, Mart A F J van de Laar

**Affiliations:** 1 Department of Psychology, Health and Technology, University of Twente, PO Box 217, Enschede, AE 7500, The Netherlands; 2 Department of Rheumatology and Clinical Immunology, Medical Spectrum Twente, Enschede, The Netherlands; 3 Department of Rheumatology, Medical Center Leeuwarden, Leeuwarden, The Netherlands; 4 Department of Rheumatology, Rijnstate Hospital, Arnhem, The Netherlands; 5 Department of Rheumatology, Gelderse Vallei Hospital, Ede, The Netherlands; 6 Department of Rheumatology, Radboud University Nijmegen Medical Centre, Nijmegen, The Netherlands

## Abstract

**Background:**

The aim of this study was to examine the measurement properties of the Dutch SF-36 version 2 (SF-36v2) health survey in patients with rheumatoid arthritis (RA).

**Methods:**

Scaling assumptions, internal reliability, and internal construct validity were examined using available data from 1884 RA patients included in the Dutch Rheumatoid Arthritis Monitoring (DREAM) registry. External construct validity and responsiveness to change were examined using baseline and 6-month follow-up data from a subset of 387 early RA patients participating in the DREAM remission induction cohort.

**Results:**

The individual items of the SF-36v2 adequately met scaling assumptions, although four items correlated too highly with items from different scales. Internal consistency was high for all eight scales and the physical and mental health components underlying the scales were replicated, supporting the use of the standard scoring algorithms. The SF-36v2 scales demonstrated minimal floor effects and ceiling effects were noteworthy only for the role-physical, social functioning, and role-emotional scales. Correlations with other core measures were as expected and the SF-36v2 showed excellent known-groups validity in distinguishing between patients with low or moderate-high disease activity. All scales related to physical health showed moderate to large responsiveness to change in patients that achieved low disease activity at six months.

**Conclusion:**

The SF-36v2 appears to be a psychometrically sound tool for the assessment of health-related quality of life of Dutch patients with RA.

## Background

Health-related quality of life (HRQOL) questionnaires are increasingly used to measure the impact of disease and the effects of treatment from the perspective of the patient. A well-known questionnaire for the assessment of HRQOL is the 36-Item Short-Form (SF-36) Health Survey [1]. The SF-36 is a multidimensional questionnaire that assesses eight different aspects of health. It is generic by nature which means that it, as opposed to disease-specific measures, can be used to measure and compare outcomes across different diseases and treatments. This feature has made generic measures of HRQOL increasingly popular among researchers and clinicians and the SF-36 has become the most frequently used measure across a wide range of range of conditions, including rheumatic diseases [2,3]. A review of patient-reported outcomes in recently published rheumatoid arthritis (RA) trials [4] showed that the SF-36 was used in 80% of the studies that reported the use of a HRQOL measure, while traditional disease-specific measures were used much less frequently.

The SF-36 has been extensively validated for use in both general and condition-specific populations in many languages and countries, including several studies in patients with RA [5-10]. These studies have generally shown the SF-36 to be a psychometrically sound measure of HRQOL in RA. Although generic measures are often assumed to be potentially less sensitive in detecting changes over time in specific diseases [11,12], studies in RA found that the different scales of the SF-36 were as responsive to changes over time as disease-specific measures within the same health domains [10,13-15].

In 1996, a new version of the questionnaire (SF-36v2) was introduced which included improvements in the instructions, the wording of some of the items, and the number of response options for two of the eight scales [16,17]. Several general population studies have confirmed the improved precision, reliability, and validity of the SF-36v2 over the original version [18,19]. To date, however, the psychometric properties of the SF-36v2 have not yet been thoroughly validated in RA patients. Therefore, the objective of this study was to examine the scaling assumptions, reliability, construct validity, and responsiveness of the SF-36v2 in a clinically diverse sample of Dutch patients with RA.

## Methods

### Patients and study design

Data for this study were derived from the Dutch Rheumatoid Arthritis Monitoring (DREAM) registry. The DREAM registry is an observational multicenter cohort study that monitors the course of RA patients undergoing different treatment regimens in the Netherlands [20]. Clinical, laboratory, and patient-reported outcomes are routinely collected and stored. Patient-reported outcomes are generally completed online. Within DREAM, 1884 unique patients from different hospital rheumatology clinics completed the SF-36v2 at least once. For the assessment of scaling properties and internal reliability and construct validity, the most recently completed SF-36v2 was selected from each patient, resulting in a sample of 1884 RA patients that completed the SF-36v2 between March 2005 and December 2012. The majority of the sample was female (64.0%) and mean (SD) age and disease duration of the patients at the time of completion were 58.3 (13.2) and 4.2 (7.6) years, respectively.

External construct validity and the responsiveness to change of the SF-36v2 were examined in a subset of early RA patients participating in the DREAM remission induction cohort [21]. The remission induction cohort consists of patients with early RA participating in a treat-to-target strategy aimed at achieving fast remission. The strategy has been shown to be highly effective, with the largest improvement in disease activity observed in the first 6 months of treatment [21]. Baseline and 6-month follow-up SF-36v2 data were available from 387 patients. Similar to the total DREAM sample, 62.4% of the patients in this subset was female and the mean age at baseline of the patients was 58.6 (14.1) years. Median symptom duration at baseline was 14.0 weeks.

The study protocol for the DREAM registry was submitted to the ethics committee of each participating hospital. Because the DREAM registry collects data for daily clinical practice, the ethics committees determined, in accordance with Dutch law, that no approval was required. Nonetheless, patients were fully informed and informed consent was obtained.

### Measures

The standard 4-week recall Dutch version of the SF-36v2 (QualityMetric Inc.) was used, which was developed using an extensive translation process which has its origins in the International Quality of Life Assessment (IQOLA) project approach [16,22]. This translation process consists of multiple forward and backward translations and cognitive debriefing interviews with native speakers of the target language. The SF-36v2 assesses different aspects of health represented in 8 scales: physical functioning (PF: 10 items), bodily pain (BP: 2 items), social functioning (SF: 2 items), mental health (MH: 5 items), general health (GH: 5 items), vitality (VT: 4 items), role physical (RP: 4 items), and role emotional (RE: 3 items) [16,17]. The PF items were intended to constitute a hierarchical Guttman scale, in which each item consistently decreases in severity or difficulty. All items are rated on Likert-type or frequency response scales, ranging from 3 response categories for the PF items to 6 categories for a BP item. Using the standard scoring algorithm [17], scales scores are linearly transformed to range from 0 to 100, with higher scores representing better health status. Additionally, the scale scores can be aggregated into two distinct (orthogonal) higher-order summary scores: a physical component summary (PCS) and a mental component summary (MCS). The component summary scores are standardized using normative data from the 1998 US general population with a mean score of 50 and a standard deviation of 10.

Beside the SF-36v2, several simultaneously collected clinical and patient-reported variables were used for analyses in the remission induction cohort sample. For all patients, disease activity was calculated using the Disease Activity Score 28 (DAS28) [23]. The DAS28 combines a 28 swollen joint count, a 28 tender joint count, the erythrocyte sedimentation rate (ESR), and a patient global assessment (PGA) of well-being on a visual analog scale into an overall continuous index. Total scores range between 0 and 10, with higher scores indicating higher disease activity. Validated cutoff scores have been defined for low disease activity (≤3.2), moderate disease activity (3.2-5.1) and high disease activity (>5.1) [24]. Patient-reported disability was measured with the Health Assessment Questionnaire Disability Index (HAQ-DI) on a scale from 0 to 3, in which higher scores indicate more disability [25,26].

### Statistical analyses

All analyses were performed using IBM SPSS Statistics version 20. Scaling assumptions and internal reliability of the SF-36v2 were examined in the first dataset (N = 1884) following the approach of the International Quality of Life Assessment project [27]. First, item-level descriptive statistics were used to evaluate the score distributions [17]. Next, correlations of each item with its own scale as well as with other scales were examined using a multi-trait/multi-item correlation matrix approach. For each scale, item internal consistency was considered satisfactory if items correlated ≥0.40 with their own scale after correction for item-scale overlap. Item discriminant validity was supported when an item correlated significantly higher (≥2 standard errors) with its own scale than with the other scales. Scaling success rates were calculated as the percentage of item scaling tests passed.

The reliability of the scales was calculated with Cronbach’s coefficient α and considered adequate for group-level and person-level comparisons when ≥0.70 and ≥0.90, respectively.

To test the internal construct validity of the scales and the hypothesized physical and mental dimensions of health underlying these scales, 0–100 transformed scale scores were computed and the pattern of correlations between the eight scales was examined. It was hypothesized that scales that were conceptually related (physical or mental health, respectively) would correlate substantially (r ≥0.40). High correlations (>0.70) were considered undesirable because this would question the distinctiveness of the scales. Further, the scale’s reliability estimates should be greater than the correlations with the other scales.

To examine the plausibility of the physical and mental dimensions, a principal component analysis with varimax rotation was performed. Two components were extracted, and the scale’s correlations with the rotated factors were examined [28]. Based on the measurement model of the SF-36 [16,17], the PF, RP, and BP scales were hypothesized to correlate most highly with the physical component and lowest with the mental component, whereas the MH, RE, and SF scales should correlate most highly with the mental component and lowest with the physical component. The GH and VT scales were expected to show substantial correlations with both components and the SF scale was expected to show a substantial cross-loading on the physical component.

External construct validity and responsiveness were examined using the 6-month data of patients participating in the remission induction cohort. External construct validity was examined by means of convergent/discriminant validity and known-groups validity [29]. For convergent/discriminant validity it was expected that the SF-36v2 scales related to physical health would be associated moderately (r ≥0.30) to strongly (r ≥0.60) with disability and well-being, moderately with the tender joint count, and weakly (r <0.30) or not at all with the swollen joint count and ESR. Overall, a similar hierarchy of weaker associations was expected for the GH and VT scales and mental scales. For known-groups validity it was examined whether the SF-36v2 was able to distinguish between patients with low (DAS28 ≤3.2) and moderate to high levels of disease activity. One-way analysis of variance (ANOVA) was employed to test for the statistical significance of group differences. The physical scales and PCS were expected to be most discriminative.

Responsiveness [30] of the scales and component scores was examined by their ability to detect changes between baseline and 6-month follow-up using paired t-tests. To examine the magnitude of change, standardized response means (SRMs) were calculated as the ratio of the mean change to the SD of that change for all scores in both the total sample and separately for patients who did or did not achieve low disease activity. Values of 0.20, 0.50, and 0.80 or greater were considered small, medium, and large, respectively [31]. Bootstrapping with 1000 samples was applied to obtain 95% confidence intervals (CIs) for the SRMs.

## Results

### Item-level descriptive statistics

The median time needed to complete the SF-36v2 was 5 minutes. The full range of responses to each item was observed (Additional file 1: Table S1). Item means clustering and ordering were comparable to those of the general population [17] and items within each scale had similar standard deviations. Mean item scores, especially for items addressing physical health, were generally lower than those in the general population. More difficult PF items generally had higher mean scores, confirming the Guttman-type properties of this scale.

### Scaling assumptions

Corrected correlations between the items and their hypothesized scales ranged from 0.36 to 0.91 (Additional file 2: Table S2). Except for one VT item (item 9a), all items passed the test for item internal consistency with correlations between the items and their scales ≥0.40 (Table 1). Additionally, all items from the RP, BP, RE, and MH scales passed the test for item discriminant validity with significantly higher correlations between the items and their scales than with the other scales. The other four scales all had one item that failed one or more scaling tests.

**Table 1:** Scaling assumptions of the SF-36v2 (N = 1884)

		**Range of correlations**		**Item scaling tests**	
**Scales**	**k^a^**	**Item internal consistency^b^**	**Item discriminant validity^c^**	**Item internal consistency^d^**	**Item discriminant validity^e^**
PF	10	0.58 to 0.79	0.24 to 0.64	10/10 (100%)	69/70 (98.6%)
RP	4	0.84 to 0.90	0.43 to 0.68	4/4 (100%)	28/28 (100%)
BP	2	0.81	0.36 to 0.70	2/2 (100%)	14/14 (100%)
GH	5	0.48 to 0.68	0.26 to 0.60	5/5 (100%)	31/35 (88.6%)
VT	4	0.36 to 0.69	0.22 to 0.62	3/4 (75%)	27/28 (96.4%)
SF	2	0.70	0.50 to 0.66	2/2 (100%)	13/14 (92.9%)
RE	3	0.86 to 0.91	0.39 to 0.64	3/3 (100%)	21/21 (100%)
MH	5	0.64 to 0.76	0.25 to 0.62	5/5 (100%)	35/35 (100%)

^a^Number of items; ^b^Pearson correlations between items and hypothesized scales corrected for overlap (correlation with the sum of the other items in the same scale); ^c^Pearson correlations between items and other scales; ^d^Number (%) of items out of k with correlation ≥ 0.40; ^e^Number (%) of items out of 7xk where the difference between the corrected correlation of the item with its own scale and correlation with the other scales ≥ 2SE (= 0.046). PF **=** Physical Functioning; *RP*, Role-physical; *BP*, Bodily pain; *GH*, General health; *VT*, Vitality; *SF*, Social functioning; *RE*, Role-emotional; *MH*, Mental health; *HT*, Health transition.

### Reliability and internal construct validity

Reliability estimates ranged from 0.79 for GH and VT to 0.95 for RP, exceeding the 0.70 standard for group comparisons for all scales and the 0.90 standard for individual comparisons for three scales (Table 2). Correlations between the scales ranged from 0.37 (PF and MH) to 0.71 (PF and RP), the latter being the only correlation exceeding the 0.70 limit for distinctiveness. Generally, the highest correlations between scales were observed between scales within either the physical or mental dimension, although several correlations between scales from the different dimensions were also substantial. All scales had higher correlations with themselves (Cronbach’s α) than with the other scales.

**Table 2:** Pearson correlations between the SF-36v2 scales, internal consistency estimates, and correlations with the two rotated factor components (N = 1884)

	**Observed correlations**								**Hypothesized**	**Observed correlations**	
**Scales**	**PF**	**RP**	**BP**	**GH**	**VT**	**SF**	**RE**	**MH**	**Dimension**	**Physical component**	**Mental component**
PF	(0.93)								P	0.85	0.19
RP	0.71	(0.95)							P	0.78	0.42
BP	0.63	0.69	(0.88)						P	0.83	0.22
GH	0.54	0.60	0.54	(0.79)					P/M	0.66	0.38
VT	0.57	0.67	0.60	0.62	(0.79)				M/P	0.60	0.59
SF	0.58	0.68	0.61	0.56	0.67	(0.82)			M	0.58	0.63
RE	0.47	0.64	0.45	0.46	0.55	0.61	(0.94)		M	0.33	0.79
MH	0.37	0.49	0.40	0.46	0.63	0.63	0.65	(0.87)	M	0.18	0.91

Scale internal consistency reliability (Cronbach’s α) is presented in the diagonal. *PF*, Physical functioning; *RP*, Role-physical; *BP*, Bodily pain; *GH*, General health; *VT*, Vitality; *SF*, Social functioning; *RE*, Role-emotional; *MH*, Mental health; *P*, Physical factor content; *M*, Mental factor content.

In the principal component analysis, the two factors explained 73.45% of the variance. Correlations between the scales and their rotated components confirmed the measurement model of the SF-36v2. As shown in Table 2, the physical scales had clearly higher correlations with the physical component (range: 0.78-0.84) than with the mental component (range: 0.19-0.42). The mental scales showed the opposite pattern (range of correlations with physical component: 0.18-0.58, range of correlations with mental component 0.63-0.91). GH and VT correlated substantially with both components, although the latter correlated more strongly with the physical component and less strongly with the mental component than expected. In accordance with the measurement model, the SF scale demonstrated a noteworthy cross-loading on the physical component.

### Statistics for scales and summary scores

Table 3 summarizes descriptive statistics and features of scale score distributions for the eight SF-36v2 scales and component summaries. The full range of the score distribution was observed for all scales. Scores tended to be negatively skewed for the scales measuring mental health (SF, RE, and MH), indicating distributions with more patients scoring among the more positive health states. Floor effects were negligible for all scales but notable ceiling effects were observed for the SF and RE scales, although these ceiling effects were less pronounced than in the general population [17], The mean PCS was almost one SD below the general population norm, whereas the MCS scores were similar to those for the general population.

**Table 3:** Descriptive statistics of the transformed SF-36v2 scale scores and normed summary scores (N = 1884)

**Scales**	**Range**^**a**^	**Mean**	**Median**	**SD**	**Skewness**	**Kurtosis**	**Floor (%)^b^**	**Ceiling (%)^c^**
PF	0 to 100	64.55	70.00	26.06	−0.53	−0.72	1.1	6.4
RP	0 to 100	57.92	50.00	27.44	−0.02	−0.83	2.5	15.5
BP	0 to 100	61.01	62.00	21.83	−0.09	−0.43	0.5	9.4
GH	0 to 100	54.61	52.00	19.60	0.03	−0.39	0.1	1.1
VT	0 to 100	57.96	56.25	19.54	−0.08	−0.43	0.1	2.1
SF	0 to 100	77.62	87.50	22.76	−0.85	0.07	0.5	35.8
RE	0 to 100	72.98	75.00	27.15	−0.68	−0.50	1.6	37.0
MH	0 to 100	75.92	80.00	17.92	−0.88	0.60	0.1	9.3
PCS ^d^	6 to 65	41.15	41.43	9.85	−0.24	−0.43	-	-
MCS ^d^	9 to 77	50.06	52.45	10.68	−0.79	0.26	-	-

^a^Observed scores; ^b^Percentage of respondents with worst possible score; ^c^Percentage of respondents with best possible score; ^d^The component summary scores are standardized using normative data from the 1998 US general population with a mean score of 50 and an SD of 10. *PF*, Physical functioning; *RP*, Role-physical; *BP*, Bodily pain; *GH*, General health; *VT*, Vitality; *SF*, Social functioning; *RE*, Role-emotional; *MH*, Mental health; *HT*, Health transition; *PCS*, Physical component summary; *MCS*, Mental component summary.

### External construct validity

The scales and component summaries demonstrated the expected pattern of associations with patient-reported disability and disease activity parameters (Table 4). As hypothesized, scales related to physical health generally correlated moderately to strongly with disability and well-being, moderately with number of tender joints, and weakly or not at all with the swollen joint count and ESR. A similar hierarchy of weaker associations was observed for the mental scales.

**Table 4:** Convergent/discriminant validity and known-groups validity of the SF-36v2 scales and summary scores (N = 387)

	**Spearman correlations at 6 month**					**Mean (SD) scores across levels of disease activity at 6 months**			
	**HAQ-DI**	**PGA**	**TJC**	**SJC**	**ESR**	**Low disease activity**	**Moderate-high**	**F(1, 331)**	**P**
	**(n = 378)**	**(n = 338)**	**(n = 338)**	**(n = 338)**	**(n = 337)**	**(DAS28 ≤3.2)**	**disease activity**		
						**(n = 232)**	**(n = 101)**		
PF	−0.76	−0.49	−0.36	−0.17	−0.13	73.56 (23.29)	52.87 (23.51)	55.18	<0.001
RP	−0.58	−0.54	−0.32	−0.19	0.02	65.46 (26.24)	43.19 (24.18)	53.11	<0.001
BP	−0.64	−0.65	−0.44	−0.29	−0.09	70.86 (18.39)	47.58 (17.28)	116.89	<0.001
GH	−0.43	−0.46	−0.24	−0.05	−0.08	63.41 (18.46)	48.85 (17.85)	44.60	<0.001
VT	−0.47	−0.47	−0.21	−0.06	−0.05	63.47 (19.10)	50.19 (18.51)	34.67	<0.001
SF	−0.51	−0.45	−0.24	−0.10	−0.01	83.73 (20.06)	69.93 (22.58)	30.83	<0.001
RE	−0.47	−0.44	−0.13	−0.05	−0.04	75.32 (26.20)	58.66 (29.20)	26.52	<0.001
MH	−0.34	−0.32	−0.13	−0.05	−0.00	77.84 (17.99)	70.00 (20.00)	12.49	<0.001
PCS	−0.71	−0.58	−0.44	−0.24	−0.12	45.77 (8.40)	36.48 (7.45)	91.97	<0.001
MCS	−0.33	−0.34	−0.07	−0.00	−0.05	50.72 (10.51)	46.16 (12.05)	12.07	0.001

*PF*, Physical functioning; *RP*, Role-physical; *BP*, Bodily pain; *GH*, General health; *VT*, Vitality; *SF*, Social functioning; *RE*, Role-Emotional; *MH*, Mental health; *HT*, Health transition; *PCS*, Physical component summary; *MCS*, Mental component summary; *HAQ-DI*, Health assessment questionnaire disability index; *PGA*, Patient global assessment of well-being; visual analog scale for general health; *TJC*, Tender joint count; *SJC*, Swollen joint count; *ESR*, Erythrocyte sedimentation rate.

Scores on all scales and the components were significantly higher for people with low disease activity, supporting strong known-groups validity of the SF-36v2 (Table 4). The physical scales and PCS were most discriminative. The PF scale performed somewhat worse than the disease-specific HAQ-DI, which obtained an F-value of 84.55 in distinguishing between the disease activity groups.

### Responsiveness to change

Mean scores on the DAS28 improved from 4.42 (1.47) at baseline to 2.73 (1.15) after six months. All eight scales and the PCS significantly improved between baseline and six months (Table 5). SRMs in the total sample were moderate for the BP scale and PCS. In the group achieving low disease activity, SRMs were at least moderate for all physical scales and large for the BP scale and PCS. All SRMs were small in the group that did not achieve low disease activity. The PF scale was slightly less responsive than the disease-specific HAQ-DI which had an SRM of 0.56 (95% CI: 0.47 to 0.66) in the total group and 0.75 (95% CI: 0.62 to 0.88) in the low disease activity group. However, these differences in SRMs were not significant in either the total group (mean difference 0.08; 95% CI: -0.01 to 0.18) or the low disease activity group (mean difference 0.03; 95% CI: -0.08 to 0.15).

**Table 5:** Responsiveness of the SF-36v2 scales and summary scores (N = 387)

				**SRM (95% CI)**		
**Scales**	**Baseline**	**6-month change**	**P**	**Total sample**	**Low disease activity**	**Moderate-high**
					**(DAS28 ≤3.2)**	**disease activity**
	**Mean (SD)**	**Mean (SD)**				
					**(n = 232)**	**(n = 101)**
PF	57.05 (25.30)	10.10 (21.47)	<0.001	0.47 (0.37 to 0.58)	0.72 (0.58 to 0.85)	0.19 (0.00 to 0.38)
RP	45.45 (27.41)	12.90 (27.01)	<0.001	0.47 (0.36 to 0.58)	0.66 (0.53 to 0.79)	0.20 (0.01 to 0.38)
BP	45.16 (21.10)	18.20 ( 24.61)	<0.001	0.76 (0.65 to 0.85)	1.07 (0.94 to 1.19)	0.40 (0.22 to 0.58)
GH	55.60 (18.30)	2.76 (16.41)	0.001	0.17 (0.05 to 0.27)	0.40 (0.27 to 0.52)	−0.21 (0.41 to −0.00)
VT	53.60 (20.61)	5.56 ( 17.72)	<0.001	0.31 (0.21 to 0.42)	0.53 (0.39 to 0.66)	0.02 (−0.17 to 0.22)
SF	70.99 (24.43)	8.04 ( 24.08)	<0.001	0.33 (0.22 to 0.44)	0.52 (0.39 to 0.65)	0.11 (−0.11 to 0.33)
RE	66.88 (28.35)	3.47 (26.79)	0.011	0.13 (0.03 to 0.23)	0.31 (0.18 to 0.44)	−0.19 (−0.40 to 0.02)
MH	71.62 (19.22)	3.88 (17.68)	<0.001	0.22 (0.12 to 0.33)	0.37 (0.23 to 0.50)	0.05 (−0.16 to 0.27)
PCS	37.13 (9.18)	5.62 (8.58)	<0.001	0.66 (0.55 to 0.76)	0.93 (0.80 to 1.07)	0.32 (0.14 to 0.50)
MCS	48.33 (11.64)	0.98 (9.95)	0.052	0.10 (0.00 to 0.21)	0.26 (0.12 to 0.39)	−0.15 (−0.36 to 0.07)

*PF*, Physical functioning; *RP*, Role-physical; *BP*, Bodily pain; *GH*, General health; *VT*, Vitality; *SF*, Social functioning; *RE*, Role-emotional; *MH*, Mental health; *HT*, Health transition; *PCS*, Physical component summary; *MCS*, Mental component summary; *SRM*, Standardized response mean.

## Discussion

This is the first study to examine the measurement properties of the generic SF-36v2 in Dutch patients with RA. The scaling assumptions of the SF-36v2 were generally supported and the questionnaire demonstrated internal reliability and internal construct validity similar to those found in the general US population. The individual scales and components demonstrated the expected pattern of associations with patient-reported and clinical outcome measures and were able to discriminate well between patients with low and moderate to high levels of disease activity. Especially the physical scales were adequately responsive to changes in disease activity. Overall, the findings suggest that the SF-36v2 is a psychometrically robust measure of HRQOL in Dutch patients with RA.

Excellent scaling success rates were found for four of the SF-36 scales (RP, BP, RE, and MH), which corresponds with findings from the original SF-36 version in the general Dutch population and in chronic disease populations [32]. All items of the SF-36v2 passed the test for item internal consistency, except for item 9a (Did you feel full of life?). This item correlated too weakly with the other vitality items and slightly more strongly with the mental health scale. Although this finding is not too surprising given the item phrasing, it has not been reported in previous studies. Given that the overall internal consistency of the vitality scale was acceptable, however, it did not substantially affect the performance of this scale. The finding that the overall general health item (item 1) also correlated substantially with several other scales corresponds with previous studies in specific patient samples [9,32,33] which also showed the lowest percentage of scaling successes for item discriminant validity of the GH scale in these populations. Despite these deviations, all eight scales met the internal reliability standards required for comparing groups of patients, and the physical function, role-physical, and role-emotional scales appear to be suitable for monitoring individuals.

In general, the observed high percentage of scaling successes lends strong support to the hypothesized scale structure of the SF-36v2 in patients with RA. The internal construct validity was further supported by the scales’ correlations with the physical and mental components of health. Principal component analysis supported the existence of the two hypothesized dimensions underlying the SF-36v2. Together the two dimensions accounted for a significant proportion (73.45%) of the reliable variance in the eight scale scores. The correlations of the scales with their principal components were as expected and were fairly similar to the hypothesized measurement model of the SF-36v2 in the general population [16,17] and those found for the original SF-36 in previous studies in patients with RA [7,9].

The vitality scale, however, correlated evenly strong with both components, whereas it correlated most strongly with mental health in the general population. Apparently, vitality is closely related to the other physical problems associated with RA, such as pain and physical functioning, a finding that is supported by the recent attention focused on the issue of increased fatigue in RA [34-36]. Similar problems with the vitality scale have also been observed in patients with severe functional somatic syndromes [33] and in people with ischemic stroke [37]. Other studies have also challenged the assumption that the way in which the eight scales relate to the physical and mental component is uniform across both diseased and healthy individuals. Findings from these studies generally suggest that the vitality scale in particular may relate to physical and mental health differently, depending upon whether a patient’s main condition is a physical or mental illness [38]. The finding that all other scales were associated with the two dimensions as expected and the high percentage of scaling successes for all scales, however, does support the legitimacy of generating scores for the eight scales and two summary measures using the standard algorithms. Moreover, using the standard US-based scoring algorithm, the PCS and MCS were negligibly correlated (r = 0.16), further supporting the orthogonal nature of the US-based component summary scores.

One of the aims of the developers of the SF-36v2 was to increase the internal reliability and to reduce the floor and ceiling effects that have been reported in the literature for the role-emotional and role-physical scales by increasing the number of response options for these scales from two to five [16]. The findings in this study suggest that these scales are indeed more reliable than in the previous version [16,32] and that especially their floor effects have been strongly reduced. Both role scales and the social functioning scale still demonstrated substantial ceiling effects, although these were much smaller than those observed in the general population [17,18]. These improvements are likely to have increased the ability of the SF-36v2 scale to discriminate between groups and to detect changes over time as compared with the original version.

The SF-36v2 demonstrated excellent convergent/discriminant and known-groups validity. The DREAM registry data allowed for a direct comparison of SF-36v2 scores with simultaneously collected self-reported and clinical core disease activity parameters [39]. The different scales of the SF-36v2 correlated as expected with the core measures of disease activity. All scales were additionally able to distinguish between patients with low disease activity and those with moderate to high disease activity as measured with the DAS-28. The DAS-28 is currently the standard-of-care measure of disease activity in RA [40] and the best determinant of the physician’s clinical judgment of response to treatment [41]. As expected, the physical scales, including bodily pain, were most discriminative. However, the physical functioning scale did not perform as well as the HAQ-DI, which over the years has become the standard measure of self-reported disability in many rheumatic conditions [42]. The HAQ-DI was still about 53% more effective in distinguishing between known groups, a finding similar to the one recently observed in patients with gout [43].

The finding that the SF-36v2 was able to discriminate well between patients with low and moderate to high disease activity, but also to detect improvements over the first six months of treatment, suggests that it can be useful for both discriminative and evaluative purposes [11] in patients with RA. The generic nature of the SF-36v2 additionally offers the opportunity of comparing the HRQOL of RA patients with those in other rheumatic and non-rheumatic conditions and with general population norms. These advantages, however, come with a potential loss of responsiveness and relevance to specific patient groups [2,11,12]. Nevertheless, in accordance with previous studies examining the original SF-36 in RA [10,13-15], this study showed that the generic nature of the SF-36v2 did not result in substantially reduced responsiveness. Especially the physical and bodily pain scales showed moderate or large improvements in those patients achieving low disease activity. Moreover, although the disease-specific HAQ-DI had better known-groups validity than the physical functioning scale, it was only slightly and non-significantly more responsive to improvements over time.

The finding that the SF-36v2 meets psychometric criteria does not necessarily mean that the questionnaire covers all issues specifically relevant to patients with RA. For instance, the physical functioning scale of the SF-36v2 mainly covers functions related to mobility and other activities requiring the use of the lower extremities, whereas finger function is not captured at all and arm function only by three items related to daily activities [44]. A recent review further indicated that the scale has limited content validity as it has no content relevant to the assessment of domestic life [45]. Therefore, for a thorough and comprehensive assessment of health-related quality of life the common recommendation to use both disease-specific and generic measures if possible [15,46] still holds.

In this light, recent initiatives to integrate and cross-calibrate generic and disease-specific measures of health-related quality of life using applications of item response theory and computerized adaptive testing are particularly interesting. Based on existing questionnaires, the NIH Patient-Reported Outcomes Measurement Information System (PROMIS) project has developed large calibrated item banks that can be used to measure key symptoms and health concepts across a wide variety of chronic diseases and in the general population [47]. This blended approach is likely to overcome the limitations of the current generation of disease-specific and generic questionnaires and may allow for more relevant, precise, and efficient assessment of health status and comparability of experiences across diseases.

Finally, it should be noted that in the current study several comparisons were made with normative data from the US general population as no Dutch norms are currently available for version 2 of the SF-36. The US norms, however, are not necessarily generalizable to other countries or cultures. Some studies comparing the US norms with those of other countries have suggested that although the magnitude of differences is generally small, for some scales they are close to or just above the difference of 5 points considered to be clinically meaningful [48-50].

In conclusion, the SF-36v2 demonstrated adequate psychometric properties in patients with RA. Using the SF-36v2 along with disease-specific measures, will allow the identification of HRQOL issues and changes in HRQOL that are important to patients and will facilitate comparisons across different disease states.

## Competing interests

The authors declare that they have no competing interests.

## Authors’ contributions

MVDL conceived of the study. PTK performed the statistical analyses and drafted the manuscript. HV, ET, PVR and MVDL supervised the study and the interpretation of the results. LS, LH, ADJ and ED critically reviewed and revised the manuscript. All authors read and approved the final manuscript.

## Supplementary Material

Additional file 1: Table S1 Distribution of responses for each item (N = 1884). Click here for file

Additional file 2: Table S2 Item descriptive statistics and Pearson item-scale correlations (N = 1884). Click here for file
