# Supplementary material for: Performance of the Dutch SF-36 version 2 as a measure of health-related quality of life in patients with rheumatoid arthritis
Source: Health Qual Life Outcomes. 2013 May 8;11:77. doi: 10.1186/1477-7525-11-77 (PMC3656790; doi:10.1186/1477-7525-11-77)
Supplement: Additional file 1: Table S1 — Distribution of responses for each item (N = 1884). [file 1477-7525-11-77-S1.doc]

**Additional file 1: Table S1:** Distribution of responses for each item (N = 1884)

|  |  | Response categories | | | | | |  |  |
| --- | --- | --- | --- | --- | --- | --- | --- | --- | --- |
| Scales | Item | 1 | 2 | 3 | 4 | 5 | 6 | Mean* | SD |
| Physical Functioning | 3a | 957 | 760 | 167 | - | - | - | 1.58 | 0.65 |
|  | 3b | 284 | 1007 | 593 | - | - | - | 2.16 | 0.66 |
|  | 3c | 298 | 971 | 615 | - | - | - | 2.17 | 0.68 |
|  | 3d | 293 | 706 | 885 | - | - | - | 2.31 | 0.73 |
|  | 3e | 137 | 597 | 1150 | - | - | - | 2.54 | 0.63 |
|  | 3f | 387 | 910 | 587 | - | - | - | 2.11 | 0.71 |
|  | 3g | 419 | 634 | 831 | - | - | - | 2.22 | 0.79 |
|  | 3h | 216 | 488 | 1179 | - | - | - | 2.51 | 0.69 |
|  | 3i | 119 | 423 | 1342 | - | - | - | 2.65 | 0.60 |
|  | 3j | 62 | 516 | 1306 | - | - | - | 2.66 | 0.54 |
| Role-Physical | 4a | 96 | 287 | 626 | 412 | 463 | - | 3.46 | 1.16 |
|  | 4b | 123 | 366 | 597 | 391 | 407 | - | 3.31 | 1.20 |
|  | 4c | 115 | 331 | 680 | 371 | 387 | - | 3.31 | 1.16 |
|  | 4d | 164 | 378 | 640 | 348 | 354 | - | 3.19 | 1.21 |
| Bodily Pain | 7 | 14 | 136 | 520 | 561 | 464 | 189 | 4.00 | 1.13 |
|  | 8 | 44 | 138 | 428 | 809 | 465 | - | 3.80 | 0.97 |
| General Health | 1 | 51 | 535 | 1030 | 209 | 59 | - | 2.84 | 0.78 |
|  | 11a | 86 | 229 | 648 | 421 | 500 | - | 3.54 | 1.14 |
|  | 11b | 145 | 478 | 497 | 553 | 211 | - | 3.11 | 1.14 |
|  | 11c | 48 | 323 | 1037 | 261 | 215 | - | 3.14 | 0.92 |
|  | 11d | 208 | 551 | 251 | 729 | 145 | - | 3.03 | 1.20 |
| Vitality | 9a | 72 | 139 | 383 | 901 | 389 | - | 3.74 | 0.99 |
|  | 9e | 75 | 322 | 570 | 767 | 150 | - | 3.32 | 0.98 |
|  | 9g | 55 | 269 | 760 | 438 | 362 | - | 3.42 | 1.04 |
|  | 9i | 177 | 494 | 842 | 269 | 102 | - | 2.80 | 0.98 |
| Social Functioning | 6 | 26 | 83 | 184 | 573 | 1018 | - | 4.31 | 0.92 |
|  | 10 | 29 | 148 | 539 | 441 | 727 | - | 3.90 | 1.06 |
| Role-Emotional | 5a | 57 | 165 | 396 | 435 | 831 | - | 3.96 | 1.13 |
|  | 5b | 85 | 194 | 425 | 415 | 765 | - | 3.84 | 1.19 |
|  | 5c | 56 | 152 | 416 | 459 | 801 | - | 3.95 | 1.11 |
| Mental Health | 9b | 18 | 70 | 415 | 647 | 734 | - | 4.07 | 0.92 |
|  | 9c | 9 | 49 | 265 | 488 | 1073 | - | 4.36 | 0.85 |
|  | 9d | 45 | 120 | 351 | 1044 | 324 | - | 3.79 | 0.89 |
|  | 9f | 22 | 74 | 461 | 566 | 761 | - | 4.05 | 0.95 |
|  | 9h | 30 | 73 | 333 | 1026 | 422 | - | 3.92 | 0.83 |
| Health Transition | 2 | 80 | 416 | 835 | 312 | 241 | - | 3.12 | 1.03 |

*Recoded so that for all items higher values indicate fewer limitations or better health.
