# Supplementary material for: Performance of the Dutch SF-36 version 2 as a measure of health-related quality of life in patients with rheumatoid arthritis
Source: Health Qual Life Outcomes. 2013 May 8;11:77. doi: 10.1186/1477-7525-11-77 (PMC3656790; doi:10.1186/1477-7525-11-77)
Supplement: Additional file 2: Table S2 — Item descriptive statistics and Pearson item-scale correlations (N = 1884). [file 1477-7525-11-77-S2.doc]

**Additional file 2: Table 2:** Item descriptive statistics and Pearson item-scale correlations (N = 1884)

|  |  | Pearson item-scale correlations* | | | | | | | |
| --- | --- | --- | --- | --- | --- | --- | --- | --- | --- |
| Scales |  | PF | RP | BP | GH | VT | SF | RE | MH |
| Physical Functioning | 3a | 0.58 | 0.55 | 0.49 | 0.46 | 0.43 | 0.39 | 0.31 | 0.24 |
|  | 3b | 0.75 | 0.64 | 0.56 | 0.47 | 0.50 | 0.53 | 0.41 | 0.31 |
|  | 3c | 0.72 | 0.61 | 0.56 | 0.45 | 0.49 | 0.52 | 0.42 | 0.33 |
|  | 3d | 0.77 | 0.55 | 0.48 | 0.45 | 0.46 | 0.45 | 0.37 | 0.29 |
|  | 3e | 0.76 | 0.51 | 0.45 | 0.38 | 0.41 | 0.45 | 0.38 | 0.29 |
|  | 3f | 0.70 | 0.55 | 0.50 | 0.42 | 0.46 | 0.44 | 0.36 | 0.29 |
|  | 3g | 0.79 | 0.58 | 0.51 | 0.47 | 0.49 | 0.46 | 0.38 | 0.29 |
|  | 3h | 0.78 | 0.53 | 0.47 | 0.40 | 0.43 | 0.46 | 0.37 | 0.30 |
|  | 3i | 0.72 | 0.48 | 0.41 | 0.34 | 0.37 | 0.41 | 0.35 | 0.26 |
|  | 3j | 0.64 | 0.51 | 0.46 | 0.35 | 0.39 | 0.44 | 0.35 | 0.28 |
| Role-Physical | 4a | 0.63 | 0.84 | 0.61 | 0.53 | 0.61 | 0.62 | 0.61 | 0.45 |
|  | 4b | 0.64 | 0.88 | 0.63 | 0.57 | 0.64 | 0.65 | 0.61 | 0.48 |
|  | 4c | 0.68 | 0.90 | 0.64 | 0.55 | 0.61 | 0.63 | 0.58 | 0.43 |
|  | 4d | 0.68 | 0.88 | 0.67 | 0.57 | 0.63 | 0.64 | 0.58 | 0.44 |
| Bodily Pain | 7 | 0.58 | 0.61 | 0.81 | 0.51 | 0.56 | 0.54 | 0.39 | 0.36 |
|  | 8 | 0.62 | 0.70 | 0.81 | 0.51 | 0.59 | 0.62 | 0.46 | 0.40 |
| General Health | 1 | 0.55 | 0.59 | 0.60 | 0.60 | 0.58 | 0.56 | 0.46 | 0.45 |
|  | 11a | 0.32 | 0.37 | 0.26 | 0.49 | 0.37 | 0.36 | 0.32 | 0.30 |
|  | 11b | 0.36 | 0.39 | 0.35 | 0.60 | 0.41 | 0.36 | 0.27 | 0.29 |
|  | 11c | 0.32 | 0.37 | 0.32 | 0.48 | 0.38 | 0.31 | 0.27 | 0.30 |
|  | 11d | 0.45 | 0.51 | 0.50 | 0.68 | 0.55 | 0.48 | 0.38 | 0.40 |
| Vitality | 9a | 0.22 | 0.28 | 0.25 | 0.30 | 0.36 | 0.31 | 0.30 | 0.40 |
|  | 9e | 0.51 | 0.59 | 0.52 | 0.55 | 0.69 | 0.58 | 0.46 | 0.54 |
|  | 9g | 0.52 | 0.62 | 0.55 | 0.53 | 0.68 | 0.62 | 0.52 | 0.54 |
|  | 9i | 0.53 | 0.61 | 0.56 | 0.55 | 0.69 | 0.58 | 0.45 | 0.48 |
| Social Functioning | 6 | 0.52 | 0.60 | 0.57 | 0.50 | 0.60 | 0.70 | 0.53 | 0.55 |
|  | 10 | 0.55 | 0.66 | 0.55 | 0.53 | 0.63 | 0.70 | 0.59 | 0.60 |
| Role-Emotional | 5a | 0.44 | 0.61 | 0.42 | 0.43 | 0.53 | 0.59 | 0.89 | 0.64 |
|  | 5b | 0.47 | 0.63 | 0.45 | 0.45 | 0.54 | 0.60 | 0.91 | 0.62 |
|  | 5c | 0.44 | 0.59 | 0.39 | 0.42 | 0.50 | 0.55 | 0.86 | 0.59 |
| Mental Health | 9b | 0.28 | 0.35 | 0.25 | 0.33 | 0.42 | 0.44 | 0.51 | 0.65 |
|  | 9c | 0.32 | 0.41 | 0.33 | 0.36 | 0.49 | 0.56 | 0.59 | 0.75 |
|  | 9d | 0.31 | 0.42 | 0.38 | 0.41 | 0.57 | 0.51 | 0.47 | 0.64 |
|  | 9f | 0.33 | 0.45 | 0.36 | 0.40 | 0.55 | 0.56 | 0.62 | 0.76 |
|  | 9h | 0.25 | 0.33 | 0.30 | 0.36 | 0.50 | 0.47 | 0.42 | 0.65 |
| Health Transition | 2 | -0.31 | -0.35 | -0.45 | -0.34 | -0.34 | -0.32 | -0.25 | -0.26 |

*Underlined correlations are corrected for overlap (correlation with the sum of the other items in the same scale).
